# Use of Handheld Raman Spectroscopy for Intraoperative Differentiation of Normal Brain Tissue From Intracranial Neoplasms in Dogs

**DOI:** 10.3389/fvets.2021.819200

**Published:** 2022-01-26

**Authors:** Caitlin E. Doran, Chad B. Frank, Stephanie McGrath, Rebecca A. Packer

**Affiliations:** ^1^Department of Clinical Sciences, College of Veterinary Medicine and Biomedical Sciences, Colorado State University, Fort Collins, CO, United States; ^2^Department of Microbiology, Immunology, Pathology, College of Veterinary Medicine and Biomedical Sciences, Colorado State University, Fort Collins, CO, United States

**Keywords:** neoplasia, brain surgery, brain shift, craniotomy, dog, meningioma, glioma

## Abstract

The aim of this study was to assess feasibility and accuracy of a hand-held, intraoperative Raman spectroscopy device as a neuronavigation aid to accurately detect neoplastic tissue from adjacent normal gray and white matter. Although Raman spectra are complicated fingerprints of cell signature, the relative shift corresponding to lipid and protein content (2,845 and 2,930 cm^−1^, respectively), can provide a rapid assessment of whether tissue is normal white or gray matter vs. neoplasia for real-time guidance of tumor resection. Thirteen client-owned dogs were initially enrolled in the study. Two were excluded from final analysis due to incomplete data acquisition or lack of neoplastic disease. The diagnoses of the remaining 11 dogs included six meningiomas, two histiocytic sarcomas, and three gliomas. Intraoperatively, interrogated tissues included normal gray and/or white matter and tumor. A total of five Raman spectra readings were recorded from the interrogated tissues, and samples were submitted for confirmation of Raman spectra by histopathology. A resultant total of 24 samples, 13 from neoplastic tissue and 11 from normal gray or white matter, were used to calculate sensitivity and specificity of Raman spectra compared to histopathology. The handheld Raman spectroscopy device had sensitivity of 85.7% and specificity of 90% with a positive predictive value of 92.3% and negative predictive value of 81.6%. The Raman device was feasible to use intraoperatively with rapid interpretation of spectra. Raman spectroscopy may be useful for intraoperative guidance of tumor resection.

## Introduction

Intracranial neoplasms have an incidence rate of 2–4.5% in canine patients, although it is widely considered that the true incidence is under-reported (1). When feasible, surgical excision can improve clinical signs as well as overall survival times (2–4). Tumor recurrence, however, is common and most frequently occurs at the resection cavity due to residual tumor volume (5).

Advances in surgical techniques and neuronavigation systems have been a focus of research in many institutions to both guide surgical planning and to reduce residual tumor burden. Neuronavigation systems, through pre-operative image acquisition, allow better visualization of tumor location and margins as well as serve as a guide for surgical planning (6, 7). Reliance on pre-operative imaging, however, has many limitations, namely, the inability to correct for brain shift (6, 8). Brain shift is the spatiotemporal phenomenon in which the positioning of the brain changes in relation to preoperative images; it is a dynamic process that is continually occurring to varying degrees with multifactorial influences including physical factors (gravity, patient positioning, device used), surgical factors (blood and CSF loss, tissue loss), and biological factors (tumor type, mannitol use) (6). The continuous dynamic nature of brain shift supports the need for real-time, intra-operative modalities to correct for this phenomenon and aid the surgeon in acquiring the most accurate margins (5, 6). Numerous techniques and intraoperative imaging modalities have been investigated to correct for brain shift, including the use of intraoperative magnetic resonance imaging (MRI) and ultrasound. While these modalities are utilized in some settings, they are limited by cost and increased surgical time for the former and difficulty aligning different image modalities and difficulty distinguishing tumor transition zones from normal brain parenchyma for the latter (6, 8).

Newer methods to correct for brain shift and aid in assessing tumor margins have gained traction in human neurosurgery. Raman spectroscopy (RS) has been used for many years in human medicine to differentiate cancerous tissues from normal tissues in cases of skin, breast, gastrointestinal, cervical, and urogenital cancers (9), but in recent years, the use of RS for intracranial tumors has increased. RS is a laser-based technology that uses light to cause a change in vibrational frequency that is interpreted through a transducer. Peaks correspond to specific vibrational modes produced by chemical bonds and can therefore be used as a means of chemical identification. The “vibrational fingerprint” of different biological tissues is created by the components of nucleic acids, lipids, and proteins (5–11). Numerous *ex vivo* studies have been performed using RS where RS was found to have >90% accuracy in identifying tumor from normal brain tissue (12–16). There are limited reports of *in vivo* use but accuracy approaches that of *ex vivo* studies (10, 17–19).

The objective of this study was to confirm feasibility of use of intraoperative handheld RS as well as to determine the sensitivity, specificity, and accuracy of RS compared to histopathology in differentiating canine intracranial neoplasms from normal brain tissue. We hypothesized that RS would not only be feasible for intraoperative use but would have a high accuracy, comparable to what is reported in human literature.

## Materials and Methods

### Case Selection

Study population consisted of canine patients that presented to Colorado State University (CSU) Veterinary Teaching hospital between August 2016 and December 2018 with clinical signs consistent with prosencephalic or multifocal disease and MRI findings consistent with a frontal or rostrotentorial mass. The prospective study was conducted in accordance with approval by the CSU Institutional Animal Care and use Committee (Protocol 14-5222A, Protocol 17-7299A, Protocol #238, Protocol #1640), and owner informed consent was obtained for all enrolled cases. Inclusion criteria included masses amenable to a transfrontal or rostrotentorial surgical approach and owner election of surgical debulking as part of the treatment process. Cases were excluded if the mass location was too deep to be grossly visualized during surgery, therefore negating the ability to visually confirm precise tissue reading location of the handheld Raman device. All cases underwent surgical planning with BrainSight Vet 2 Neuronavigational system (Rogue Research, Inc., Montreal, Quebec, Canada). The BrainSight system was used to help plan sample acquisition points. Where feasible, four tissue areas were interrogated with RS, and biopsy of that interrogated area was submitted for histopathology. Planned areas included: brain tumor core, normal white matter, normal gray matter, and brain tumor edge, when available. If all planned locations were not easily accessible or visible intraoperatively, only those that could be obtained without additional patient detriment were interrogated and acquired.

### Raman Spectroscopy Device and Acquisition Procedure

The Raman spectroscopy machine (Synaptive Medical Inc., Toronto, Ontario, Canada) consisted of a lightbox, computer laptop, and foot pedal which were kept on a rolling cart with an ~2 × 2-ft footprint. The probe tip of the RS had an ~2–3-mm diameter surface area which needed to be in direct contact to the tissue to be interrogated in order to acquire an adequate signal.

### Raman Spectra Collection Procedure

Patients were placed in sternal recumbency and prepped for surgery as standard for rostrotentorial or transfrontal approach. The surgical procedure continued routinely until the tumor was visible, at which time RS acquisition occurred. For each of the planned acquisition sites (brain tumor core, normal white matter, normal gray matter, or brain tumor transition zone), the RS probe was placed in direct contact with tissue in the target areas, while the neuronavigation system was used to confirm the target location used for signal acquisition. Following acquisition, the probe was removed from the site and a 2-mm biopsy of the interrogated tissue was collected and labeled based on RS interpretation and submitted for histopathology. The planned sample sites were only performed if the site was easily accessible intraoperatively. Once all RS samples were collected, the mass was debulked in standard fashion and the surgical site was routinely closed.

During Raman spectra acquisition the surgical and room lighting were turned off and lights from surgical loupes were directed away from the surgical site. The handheld RS probe was held perpendicularly in full contact with the tissue to be interrogated, while the assistant acquired the spectra readings using the computer console. A foot pedal served as a laser safety device, and the laser would not deploy or would automatically cease if the surgeon stopped depressing the foot pedal. Acquisition parameters were optimized prior to initiation of the study and set consistently at 0.5 s × 10 averages per acquisition for all patients. A total of 5 readings were acquired from each site and analyzed on screen intraoperatively to assess the degree of shift of the lipid-to-protein ratio to determine if the interrogated tissue was neoplastic vs. normal white or gray matter. Each reading was assigned either a 1 or 0 for neoplastic or normal brain tissue, respectively. Interrogated areas with at least one neoplastic reading out of the five were deemed neoplastic and those with zero of the five readings as neoplastic were deemed normal brain tissue.

### Histopathology

Samples collected during surgery were fixed in 10% neutral buffered formalin, embedded in paraffin, section at 5 μm, and stained with hematoxylin and eosin. The pathologist assessed the entire biopsy sample and established a percentage of neoplastic cells of the entire sample from 0 to 100. Additional immunohistochemical staining was at the discretion of the pathologist to diagnose tumor type.

### Statistical Analysis

Raman spectroscopy was compared to histopathology as the gold standard to calculate sensitivity, specificity, positive, and negative predictive value, as well as accuracy of the RS probe in differentiating neoplastic from normal brain tissue. A 2 × 2 tablet and Microsoft Excel (Microsoft Excel 2013) was used for statistical calculations.

## Results

### Cases

A total of thirteen dogs were enrolled in the study and consisted of 8 spayed female and 5 neutered male dogs. Breeds enrolled included three Golden Retrievers; two Boxers; two Australian Shepherds; two Maltese/Maltese mixes; and one each of German Shepherd, Border Collie, Labrador mix, and mixed-breed dog. Dogs weighed an average of 21.2 kg and a median of 27.6 kg (range 4–31.8 kg) and were an average of 7.9 years of age (range 5–13 years). Seven out of 13 dogs had left-sided masses and the remaining six were right sided. Masses were most commonly located in the olfactory and frontal lobes (4/13), followed by the frontal and parietal lobes (2/13), parietal lobe (2/13), frontal lobe (2/13), frontal and temporal lobes (1/13), temporal lobe (1/13), and occipital lobe (1/13). Six dogs underwent a transfrontal or modified transfrontal craniectomy, and the remaining 7 dogs had a rostrotentorial craniectomy. Masses were diagnosed as meningioma (7/13), histiocytic sarcoma (2/13), and one each of fungal granuloma, high-grade oligodendroglioma, low-grade oligodendroglioma, and high-grade astrocytoma. Results from the fungal granuloma case were excluded from the study as were results from one dog with a meningothelial meningioma in which acquisition of data was not obtained due to intraoperative complications unrelated to the study. Of the remaining 11 dogs, 24 Raman and histopathological samples were obtained, 13 of which comprised neoplastic tissue, and 11 of which were normal gray or white matter. Although a total four sites were initially planned per dog, samples could only be acquired when easily visible in the surgical field and therefore not all planned samples were obtained.

### Raman Spectroscopy and Histopathology Data

A total of five Raman spectroscopy readings were obtained from each site with each waveform being analyzed real time. Each site was assigned a value of 0–5 out of 5 readings consistent with neoplastic tissue. Of the 24 histopathologic samples, 14 samples (58%) contained >1 percent neoplastic population and were therefore considered neoplastic samples; 9 of the 14 comprised a 100% neoplastic population. The remaining 10 histopathology samples contained 0 percent neoplastic cells and were therefore considered normal brain tissue.

**Figure 1:**
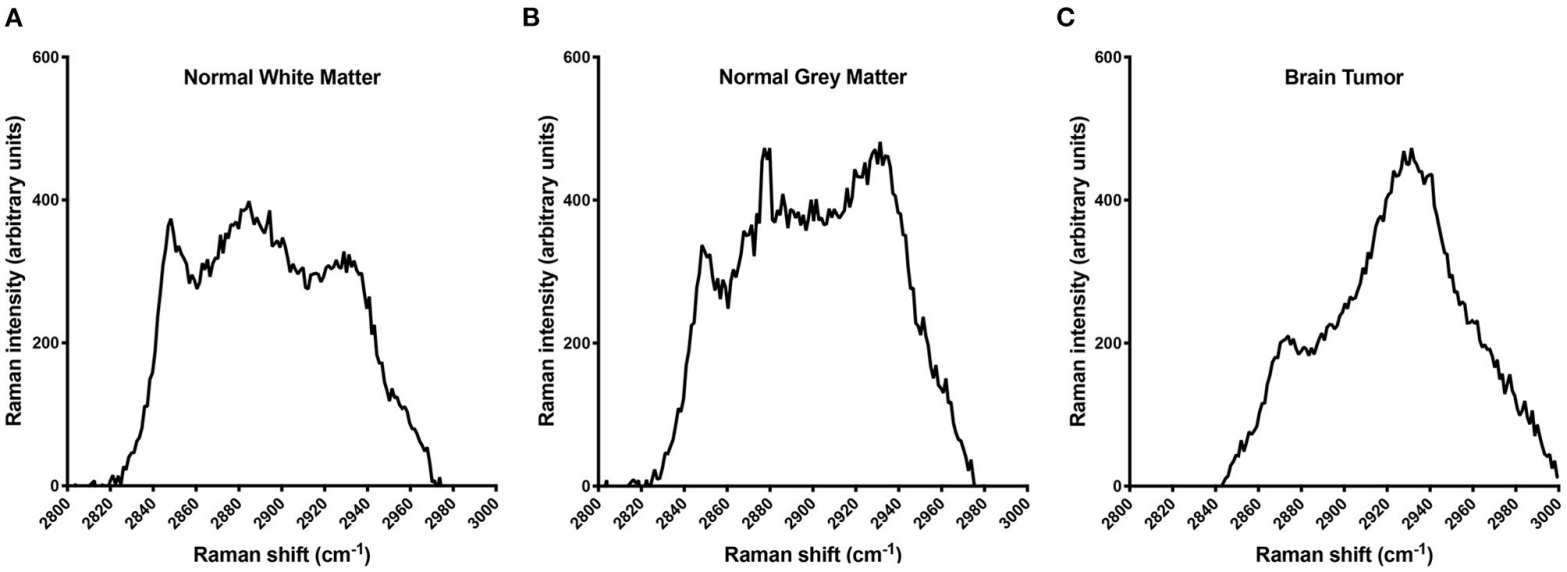
Raman spectra illustrating the differences in the protein:lipid ratio among normal white matter **(A)**, normal gray matter **(B)**, and brain tumor **(C)**. The decreasing lipid content among white matter, gray matter, and tumor tissue accounts for this shift. In a clinical setting, all five readings are used individually to screen for a protein:lipid ratio indicative of tumor tissue; however, for illustrative purposes the data presented here are averages of the five interrogations for samples 2, 5, and 6, respectively.

Individual dog results for RS and histopathology can be found in Table 1. Of the 24 Raman spectroscopy samples, 13 (54%) had between one and five out of the five readings consistent with neoplasia and were therefore considered neoplastic samples, and in nine of those 13, all five readings were consistent with neoplasia. The remaining 11 samples (46%) had zero out of five neoplastic readings and were consistent with normal white or gray matter. Representative RS waveforms and peaks for normal white matter, gray matter, and brain tumor can be found in Figure 1.

**Table 1:** Histopathologic diagnosis, Raman spectroscopy results, and histopathologically determined percent neoplastic tissue for individual samples.

**Sample number**	**Histopathological diagnosis**	**Raman spectroscopy neoplastic readings (0–5/5) and interpretation if non-neoplastic (white vs. gray matter)**	**Histopathology percent neoplastic tissue**
1	Meningioma	5/5	100% neoplasia
2	Normal white matter	0/5—White matter	0% neoplasia
3	Normal white matter	0/5—White matter	0% neoplasia
4	Meningioma	5/5	100% neoplasia
5	Normal gray matter	0/5—Gray matter	0% neoplasia
6	Meningioma	5/5	100% neoplasia
7	Glioma	1/5	15% neoplasia
8	Mixed neoplasia and normal	0/5—White matter	10% neoplasia
9	Mixed neoplasia and normal	0/5—White matter	30% neoplasia
10	Glioma	5/5	85% neoplasia
11	Glioma	5/5	100% neoplasia
12	Normal white matter	0/5—White matter	0% neoplasia
13	Histiocytic sarcoma	5/5	85% neoplasia
14	Normal white matter	0/5—White matter	0% neoplasia
14	Glioma	2/5	15% neoplasia
15	Glioma	2/5	15% neoplasia
16	Meningioma	5/5	100% neoplasia
17	Normal white matter	0/5—White matter	0% neoplasia
18	Normal white matter	0/5—White matter	0% neoplasia
19	Meningioma	5/5	100% neoplasia
20	Normal white matter	0/5—White matter	0% neoplasia
21	Meningioma	4/5	100% neoplasia
22	Normal white matter	1/5—White matter	0% neoplasia
23	Normal white matter	0/5—White matter	0% neoplasia
24	Glioma	3/5	100% neoplasia

### Statistical Analysis

Raman spectra samples with a value of 0/5 were determined to be normal brain tissues and those with values assigned between 1 and 5/5 were deemed neoplastic samples for statistical analysis. Those with a percentage of zero were deemed normal brain tissue, and those with >1% neoplastic cells were considered neoplastic samples for statistical analysis. Twelve of the 24 acquired samples (50%) were considered neoplastic by both RS and histopathology and nine of the 24 samples (37.5%) were considered normal brain tissue by both. Raman spectroscopy and histopathology did not match in three of the 24 cases (12.5%) where two of the three samples were normal on RS and neoplastic based on histopathology, and one sample was neoplastic on RS and normal tissue based on histopathology (Table 2). Raman spectroscopy had sensitivity of 85.7%, specificity of 90%, positive predictive value of 92.3%, and negative predictive value of 81%. Accuracy was calculated to be 87.5%.

**Table 2:** Two by two table of Raman spectroscopy and histopathology sample results which was utilized to calculate sensitivity, specificity, and accuracy of Raman spectroscopy compared to histopathology.

	**Histopathology, neoplastic tissue (1–100%)**	**Histopathology, normal brain tissue (0%)**
Raman spectroscopy neoplastic (1–5/5)	12	1
Raman spectroscopy normal brain tissue (0/5)	2	9

## Discussion

Handheld RS was feasible for intraoperative use in this study with sensitivity and specificity of differentiating neoplastic tissue from normal brain of 85.7 and 90%, respectively; accuracy of RS was 87.5%. Our findings are similar to what is reported in human literature, supporting our hypothesis. The majority of RS studies involving brain tumors report use in an *ex vivo* setting; however, there are limited *in vivo* reports. *Ex vivo* studies in human medicine have found very high sensitivity and specificity; RS was 97% sensitive and 98.5% specific in detecting tumor cells in one study (12) and 97.4% with specificity of 100% in differentiating glioblastoma, medulloblastoma, and meningiomas from normal tissue groups in another (14). The results of these *ex vivo* studies paved the way for *in vivo* use. In one such *in vivo* study, the ability of RS to determine tumor margins compared with MRI-determined margins was investigated, revealing RS sensitivity of 92%, specificity of 93%, and accuracy of 92% in detecting cancer cells (18). This study also found that RS was able to identify margins over 2 cm beyond what was established by MRI (18). Raman spectroscopy was utilized in another study to distinguish necrosis from tumor and normal brain finding an accuracy of 87%, 84% sensitivity, and 89% specificity (19). An additional intraoperative RS study determined RS to have sensitivity of 93%, specificity of 91%, and accuracy of 92% in differentiating normal brain tissue from cancer (10). A study assessing a combination handheld RS and biopsy instrument to target deep brain cancer tissue biopsies found RS to have 80% sensitivity, 90% specificity, and 84% accuracy in differentiating dense cancer (areas with >60% neoplastic cells) from non-diagnostic samples (those composed of normal brain or samples with <60% neoplastic cells) (20). These *in vivo* studies assessed RS use in similar fashion to our study and ranged in sensitivity from 80 to 93%, ranged in specificity from 89 to 93%, and ranged in accuracy from 84 to 92% (10, 18–20); however, the previous studies in humans used slightly different methods from our study, making direct comparison of results challenging. Overall, our results are similar to those reported in previous *in vivo* human studies, supporting the continued investigation into RS use in veterinary medicine.

We considered samples in our study to be neoplastic when histopathological examination of the biopsy revealed >1% neoplastic cells or when RS had at least one neoplastic reading. We utilized these cut offs as we were investigating RS use as a tumor resection tool to obtain the most accurate margins possible, leaving minimal residual tumor burden. Brain tumor recurrence and associated clinical signs following initial surgical debulking is well-documented in human medicine for various tumor types (21–23), but is mostly speculative in veterinary literature (25). Residual tumor burden has been shown to have an impact on survival after surgery for human glioblastoma (2, 21), and maximal resection has been the standard of care for initial treatment and recurrence of glioma (2, 23). Although infrequently confirmed by repeat imaging or post-mortem examination, it is speculated that progression of neurological signs in veterinary patients following surgery is attributable to tumor regrowth (25). In humans, meningiomas are seen to recur in 9–12% of cases with gross total resection and upwards of 40% of cases with incomplete resection (24, 25); it has been hypothesized that recurrence of meningiomas in cases with complete resection may be secondary to the presence of neoplastic cells in the adjacent nervous tissue (17, 23). To reduce tumor burden and increase time to recurrence, intraoperative RS guidance may be helpful.

The study initially planned to obtain four RS and histopathology samples from each dog. However, due to the inherent challenges of intracranial surgery, not all planned data acquisition points could be met. This limited the number of samples analyzed and could have affected the sensitivity, specificity, and accuracy found with RS. We anticipated a small sample size for this study as the overall incidence of canine brain tumors is <5% (1, 26) and only a smaller subset of those are in a rostral cranial fossa location, amenable to inclusion in this study. For routine clinical use, planned data points would not occur during tumor resection; the RS probe would instead be used to interrogate the visible surgical field, particularly the margins of the resection cavity after gross disease was resected, and help confirm that resection of microscopic disease was complete. Thus, despite our small sample size, the inability to collect all planned points did not greatly impact the RS validation for prospective intraoperative use.

Our study has several limitations, the most significant being the small sample size. Canine brain tumors have an incidence of <5% (1, 26) and fewer of those have tumors in the location desired for this study and opt for surgical intervention as part of therapy. As these were client owned animals and not part of a terminal study, the overall patient well-being was prioritized, limiting our sample acquisition. An additional limitation is that due to the RS probe and biopsy instrument being separate tools, we are unable to guarantee that the RS interrogated tissue was exactly that of what was submitted for histopathology. This may have led to some of the discrepancies in readings. However, despite these limitations, the sensitivity and specificity for RS compared to histopathology remained high. A novel RS system with the ability to biopsy following interrogation of tissue has been used in human medicine with a high sensitivity and specificity (20). Utilizing a similar combination instrument for this study that interrogates and biopsies the interrogated site could have strengthened our results and would be preferred for future studies.

Raman spectroscopy was investigated as a potential method to assist with complete neoplastic tissue resection in intracranial surgery in dogs. The feasibility of intraoperative use and high sensitivity and specificity of RS in this preliminary report support its use in future studies. Further studies are needed in veterinary medicine to compare the achievability of RS in minimizing residual tumor burden with that of more standard surgical guidance techniques.

## Data Availability Statement

The raw data supporting the conclusions of this article will be made available by the authors, without undue reservation.

## Ethics Statement

The animal study was reviewed and approved by Colorado State University Institutional Animal Care and Use Committee. Written informed consent was obtained from the owners for the participation of their animals in this study.

## Author Contributions

CF performed all histopathology readings. RP designed the study and performed raw data analysis. SM and RP were primary surgeons in all cases and collected raw data. CD compiled all spectra, performed statistical analysis, and drafted the manuscript. All authors read, revised, and approved of this manuscript for submission for publication.

## Funding

Funding for this study was provided by the Colorado State University Cancer Supercluster Grant, and the Eldred Foundation.

## Conflict of Interest

The authors declare that the research was conducted in the absence of any commercial or financial relationships that could be construed as a potential conflict of interest.

## Publisher's Note

All claims expressed in this article are solely those of the authors and do not necessarily represent those of their affiliated organizations, or those of the publisher, the editors and the reviewers. Any product that may be evaluated in this article, or claim that may be made by its manufacturer, is not guaranteed or endorsed by the publisher.

## Abbreviations

CSU: Colorado State University
MRI: Magnetic resonance imaging
RS: Raman spectroscopy.
